# Presence of SNPs in GDF9 mRNA of Iranian Afshari Sheep

**Published:** 2012-03-20

**Authors:** Shahin Eghbalsaied, Kamran Ghaedi, Somayeh Shahmoradi, Akbar Pirestani, Hamidreza Amini, Talat Saiedi, Linda Nicol, Alan McNeilly

**Affiliations:** 1 Department of Animal Science, Agricultural Faculty, Khorasgan Branch, Islamic Azad University, Isfahan, Iran; 2 Biology Department, School of Sciences, University of Isfahan, Isfahan, Iran; 3 Departments of Cell and Molecular Biology, Cell Science Research Center, Royan Institute for Animal Biotechnology, ACECR, Isfahan, Iran; 4 Young Researchers Club, Khorasgan Branch, Islamic Azad University, Isfahan, Iran.; 5 MRC Centre for Reproductive Health Queen's Medical Research Institute, University of Edinburgh, Scotland, UK

**Keywords:** GDF9, SNP, Fecundity, Sheep, Twining

## Abstract

**Background:**

Multiple births occur frequently in some Iranian sheep breeds, while infertility scarcely occurs. Mutation detection in major fecundity genes has been explored in most of Iranian sheep flocks over the last decade. However, previously reported single nucleotide polymorphisms (SNPs) for bone morphogenetic protein receptor-(BMPR)-1B and growth differentiation factor ) GDF9( known to affect fertility have not been detected. This study was conducted to assess whether any significant mutations in GDF9 were extracted from slaughtered ewe ovaries of Iranian Afshari sheep breed.

**Materials and Methods:**

Ovaries defined as poor, fair, and excellent quality based on external visual appearance of follicles were used for histology and RNA extraction processes. High quality RNAs underwent reverse transcriptase-polymerase chain reaction (RT-PCR) from GDF9 mRNA, and the products sequenced.

**Results:**

No streak ovaries, which are considered indicators of infertility due to homozygocity for some mutations in GDF9 and BMP15, were found. Sequencing results from GDF9 cDNA showed that G2 (C471T), G3 (G477A), and G4 (G721A) mutations were observed from 1, 4, and 1 out of 12 ewes, respectively. Though all 3 mutations were previously reported, this is the first report on their presence in Iranian breeds. The first and second mutations do not alter the amino acids, while G4 is a non-conservative mutation leading to E241K in the prohormone.

**Conclusion:**

As the G4 mutation was observed only in ovaries defined superficially as top quality, it could be considered as one of reasons for higher ovulation rate in some sheep. Furthermore since multiple mutations were observed in some cases, it might be possible that combinations of minor mutations in GDF9 and BMP15 interact to affect fecundity in some Iranian sheep breeds.

## Introduction

Normal expression of the oocyte specific genes growth differentiation factor-9 (GDF9), located on chromosome 5 and bone morphogenetic protein (BMP15), also known as GDF-9B , located on the X chromosome (1, 2), are necessary for normal follicle growth and development in sheep. Inactivating mutations of either or both GDF9 and BMP15 lead to infertility in homozygous ewes, but increased fertility in heterozygous ewes (3-5). The GDF9 gene includes two exons 1126 bp length and encodes a premature protein of 453 amino acids which in its mature form contains 135 amino acids (2). Moreover, the bone morphogenetic protein receptor- 1B (BMPR-1B; ALK 6) mutation induces precocious maturation of ovarian follicles by increasing the sensitivity of the follicles to follicular stimulating hormone (FSH) without an increase in FSH concentrations (6).

Even though the mutation in autosomal BMPR1B additively increases sheep fecundity, some GDF9 mutations enhance ovulation rates only in heterozygous animals (3,7). However, a recent publication indicates the presence of fertility in sheep homozygous for some mutations of either GDF9 or BMP15 genes (8). So far, eight point mutations in Belclare and Cambridge breeds (7), and one more mutation in Thoka breed (4) were reported for the GDF9 gene.

There are high variations among different Iranian sheep breeds in terms of carcass yield and prolificacy. Twin births are frequent in some breeds though infertility is rarely observed in these flocks. Iranian sheep flocks have been analysed for mutations in these major fecundity genes over the last decade but no significant mutations were detected (9). Predefined mutant alleles with either additive inheritance BMPR-1B or over-dominance manner BMP15 and GDF9 were not detected in Iranian Shaal and Ghezel breeds (9-11). However, results from a recent study indicates the presence of G1 and B2 mutations in GDF9 and BMP15 genes, respectively, in Moghani and Ghezel breed (8).

This study was performed on Iranian Afshari sheep screened GDF9 mRNA extracted from slaughtered ewe ovaries classified in terms of the degree of follicle development on external morphological appearance, and reports the presence of 3 previously known GDF9 mutations, one of which is associated with increased fertility.

## Materials and Methods

All the following procedures which were carried out on animals were approved by the Animal Welfare Committee and the Halal Commission of Khorasgan Branch, Islamic Azad University.

### Samples

Since we did not have a reliable database of sheep fecundity trait, follicular and morphological status of ewe ovaries were considered as an indicator for ovulation rate and its consequential litter size. After slaughtering 30 ewes, ovaries were placed in normal saline and transferred to the laboratory within 2-3 hours where they were classified into 3 categories based on follicle number including poor (no observable follicles on the surface), good (regular), and excellent (containing abundant follicles). Among the 30 pairs of ovaries, 10 showed excellent and another 10 showed poor follicle numbers and thus were assigned to excellent and poor groups respectively. Homozygote and heterozygote genotypes for either BMP15 or GDF9 have been considered to result in sterility and high fecundity, respectively (4, 7). Therefore, ovaries from all three groups underwent histology and mRNA sequencing for GDF9.

### Histology

Eight ovaries each from poor, good, and excellent groups were selected at random for histological evaluation. Following fixation in 10% PFA, ovaries were sectioned into 5 microns using microtome and underwent hematoxylin and eosin staining procedure to discriminate nucleus and cytoplasm. Slides were deparaffinized and rehydrated in descending graded series of alcohol and distilled water. Following hematoxylin staining, destaining was performed in acid-ethanol and distilled water. Finally, slides were stained with eosin and dehydrated in graded ethanol concentrations.

### Reverse transcriptase-PCR

Thin slices of ovaries were immediately thawed using the freeze-thawing process followed by RNA extraction in 1 ml of AccuZol (#K3090, Bioneer) and 100 μl of chloroform. The mixture was centrifuged for 15 minutes at 4°C. Equal volume of Isopropyl alcohol in addition to 1μl glycogen (RNA grade, #0551, Fermentas) were added to the supernatants and stored at -20°C for 2 hours. After removing of Isopropyl alcohol, washing steps by ethanol were repeated. The RNA pellet was air dried at 37°C for 5 minutes and dissolved in 25 μl of DEPC treated water. Presence of a unique RNA pattern on agarose gel electrophoresis indicated a high quality of extracted RNA.

Reverse transcription (RT) step was conducted using RevertAid First St cDNA kit (#EP0441, Fermentas) with minor modifications. Briefly, 1 μl (3 μg) of total RNA and 1 μl of random hexamer primers were added into 10 μl of DEPC water. The mixture was incubated at 70°C for 5 minutes, chilled on ice, and mixed with RT ingredients including 5X reaction buffer, dNTP mix (200 μM), RNase inhibitor, and RT enzyme (1 μl). The cDNA was synthesized via incubation at 25°C for 5 minutes, 42°C for 60 minutes, and 70°C for 5 minutes. One micro litre of the cDNA was used for PCR reaction to amplify a 589 bp fragment with (5'-CAACACTGTTCGGCTCTTCACC-3') and (5'-CAATTCAGAGCTGGCACTCTCC-3') as forward and reverse primers, respectively (3). A total of 25 μl PCR reaction mixture contained: 50 ng of cDNA dissolved in Diethylpyrocarbonate (DEPC) treated water, forward and reverse primers (10 pM), dNTPs mixture (200 μM each), 10X PCR buffer, 50 mM magnesium chloride, and 0.5 unit of Ex-Taq DNA Polymerase (#RR01, TaKa- Ra). PCR cycling conditions initiated at 94°C for 4 minutes, followed by 35 cycles of 94°C for 30 seconds, 59°C for 20 seconds, and 72°C 30 seconds, and ended by an extra extension at 72°C for 7 minutes. The resulting PCR products were electrophoresed on 1% agarose gel which was pre-stained with ethidium bromide (0.5 μg/ ml), and photographed using the transilluminator (UVITECH Cambridge). PCR products from 12 ewe ovaries which showed the desired banding pattern were sent for sequencing.

### Results

### Histology

Sections at different parts of each ovary from poor, fair, and excellent ovaries were used for histology. Though no follicles were detected on the surface of poor ovaries, the histology examination revealed the presence of follicles at all different stages of growth and development and there was no indication of any failure of follicle development as seen in e.g. homozygous Thoka GDF9 mutant sheep (3).

### RT-PCR and sequencing

**Fig 1:**
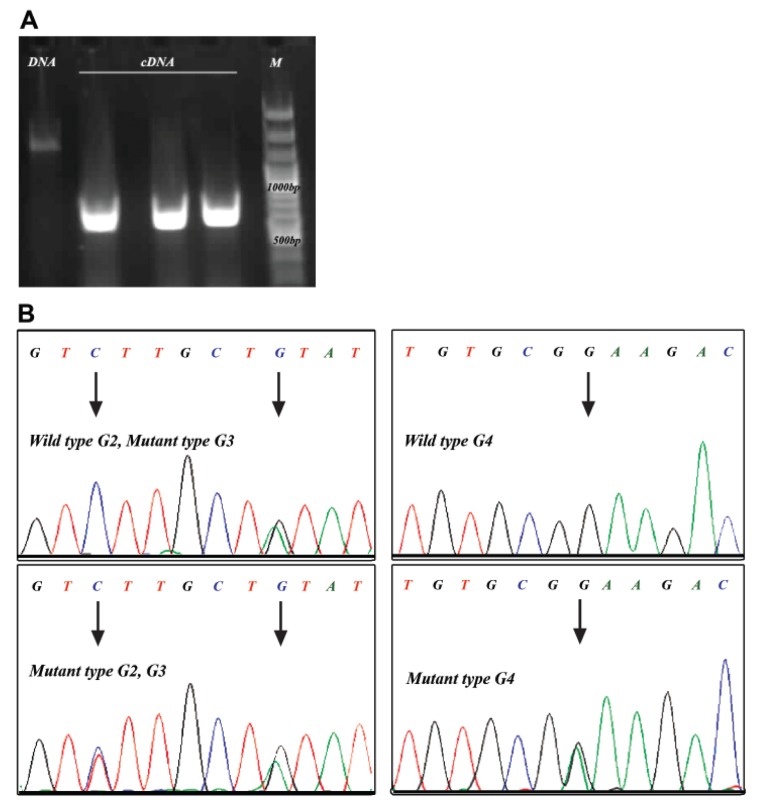
GDF9 PCR from sheep ovaries genome (DNA) and cDNA (A), and sequencing results for GDF9 mRNA showed G2, G3, and G4 mutations (B).

To determine the potential expression of GDF9 mutations among different initial classifications of ovaries, RNA extraction and reverse transcription procedures for GDF9 gene were conducted (Fig 1).

**Fig 2:**
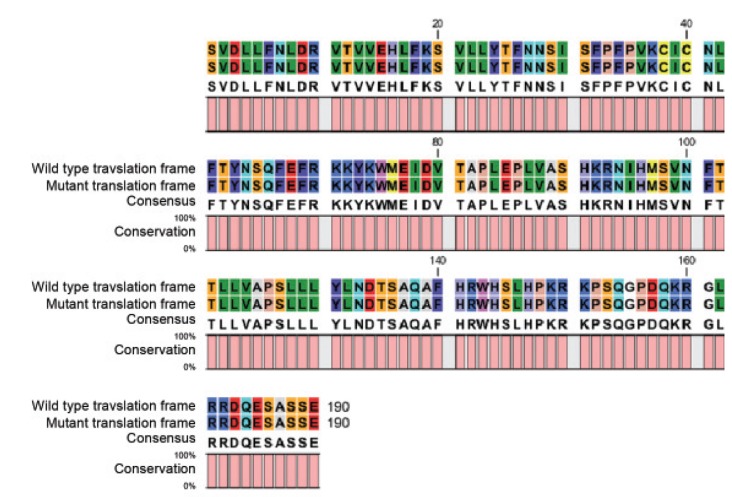
Amino acid alignment for G4 (G721A) mutation which leads to nonconservative E241K shift in unprocessed amino acid peptide.

Sequencing results from the GDF9 cDNA showed that there were 3 point mutations compared to NM_001142888 code in 1 out of 12 ovaries (Fig 1). This is the first report of presence of G2, G3, and G4 GDF9 mutations in Iranian sheep breeds. Detected sequences from forward and reverse primers coupled with the sequencing graph showed the presence of three point mutations which were confirmed by repeated sequencing. The first and second mutations at C471T and G477A did not change the amino acid polypeptide sequence. However, there was a significant shift in the amino acid sequence (E241K) due to swapping of G at base 721 with A which caused a non-conservative amino acid change (Fig 2). Frequencies of G2, G3, and G4 SNPs in 12 sequenced amplicons were 1, 4, and 1, respectively (Table 1). Interestingly only a high quality ovary contained all three mutations. All the sheep were heterozygous for the detected mutations and no homozygous cases were detected.

**Table 1:** Abundance of G2, G3, and G4 SNPs in 12 sheep mRNA


Embryo quality	Sample size	G2 (C471T)	G3 (G477A)	G4 (G721A)

**Poor**	3	0	3	0
**Fair**	5	0	0	0
**Excellent**	4	1	1	1


## Discussion

Lack of registered records for fertility traits in Iranian sheep flocks has been considered as the main obstacle for major gene detection. Our investigation of different flocks for sterile ewes indicated that repeated signs of oestrous (heat), without pregnancy is rare. However the lack of reliable breeding records resulting in uncertainty in determining high prolificacy ewes forced us to use follicle number as potential indirect sign for ovulation rate and litter size. Histological assessment of more than 10 poor ovaries indicated that there were numerous follicles at all stages of development inside white ovaries which makes them comparable in terms of follicle development to high quality ovaries. This indication from abattoir-derived ovaries plus the very low incidence of infertility suggests that significant mutations in GDF9 which led to infertility and high prolificacy in Belclare and Cambridge (7), and Thoka (4) breeds might not be the cause of the increased twining rate in Iranian breeds.

In accordance to Hanrahan et al. (7), our results showed that there are three mutations in Iranian breeds. Both the G2 (C471T) and G3 (G477A) mutations caused no substitution in the translated amino acid and were thus of no consequence. However, the G4 mutation replaces glutamic acid with lysine at amino acid residue 241 of the unprocessed protein, and leaves a basic group in place of an acidic group. Though this SNP occurs at a position before the furin processing site, it caused a non-conservative change in amino acid sequence and was considered as the second important mutation in sheep GDF9 (7) before the identification of the Thoka mutation (4). Therefore, its occurrence in high quality ovaries could be related directly to the increased fecundity in some Iranian breeds. So far, G1 has been the only GDF9 mutation in Iranian breeds (8). The G1 conservative arginine to histidine shift, which causes substitution of a basic charged polar group with another before the furin cleavage site, was assumed to have minimal or no effect on sheep fecundity (7). This hypothesis was challenged in Iranian Ghezel and Moghani breeds (8). However they did not determined the presence of the G4 SNP. Thus, the significance of the G1 mutation in the presence of the G4 or other unknown mutations still remains to be determined.

Other investigations of Iranian breeds showed that major mutations on twining rate are not the case for higher prolificacy of Iranian sheep (9-11). In summary, none of GDF9, BMP15, and BMPR- 1B detected mutations was observed in Shaal breed which had the highest litter size among Iranian sheep breeds. Neither BMP15 nor BMPR-1B mutations caused the high prolificacy of Iranian Lori-Bakhtyari and Ghezel breeds. Moreover, the G8 mutation which caused an over dominance phenotype in Belclare and Cambridge breeds was not detected in Iranian breeds (10).

So far, presence of the G1 (8) and the G4 (present study) SNPs have been reported to be associated with prolificacy in some Iranian sheep breeds. This might be due to the change in the GDF9 propeptide which could potentially act as GDF9 inhibitor (12, 13). However, assessment of G1 and G4 mutations and their combined function requires to be investigated fully to determine whether or not they are related to the high twining rates in Iranian sheep breeds.

## Conclusion

Twin births in some Iranian sheep breeds are common, though infertility is scarcely detected. Our investigation on GDF9 mRNA extracted from abattoir-derived ovaries showed that there were 3 point mutations including two conservative substitutions, G2 (C471T) and G3 (G477A), and one non-conservative mutation, G4 which replaces glutamic acid to lysine (E241K) in the unprocessed protein. Though the G4 mutation was not supposed to significantly affect prolificacy in sheep flocks having more important mutations (7), its presence in high quality ovaries in Iranian sheep might indicate its importance for twining rate. Moreover, aggregative effect of minor mutations in the whole mRNA sequence including both unprocessed and processed peptides might be the reason for relatively higher fecundity in some Iranian sheep breeds. However, screening of the complete sequences including untranslated regions (UTRs) of GDF9, BMP15, and BMPR-1B is required to determine the mechanism by which high prolificacy occurred in Iranian sheep breeds.
